# Optimizing Vegetation Restoration: A Comprehensive Index System for Reclaiming Abandoned Mining Areas in Arid Regions of China

**DOI:** 10.3390/biology14010023

**Published:** 2024-12-29

**Authors:** Aishajiang Aili, Yuguang Zhang, Tao Lin, Hailiang Xu, Abdul Waheed, Wanyu Zhao, Amannisha Kuerban, Kun Liu, Haitao Dou

**Affiliations:** 1 State Key Laboratory of Desert and Oasis Ecology, Xinjiang Institute of Ecology and Geography, Chinese Academy of Sciences, Urumqi 830011, China; aishajiang@ms.xjb.ac.cn (A.A.); wanyuzhao@ms.xjb.ac.cn (W.Z.); amannisa024@163.com (A.K.); lklyk1996@163.com (K.L.);; 2 State Investment and Development Corporation, Hami Energy Development Co., Ltd., Hami 839000, China; mining_rest@sohu.com; 3 Desert-Oasis Ecological Monitoring and Restoration Engineering Innovation Center, Ministry of Natural Resources, Urumqi 830002, China; xjlintao@hotmail.com

**Keywords:** ecological restoration, application, index system, abandoned mining area, review

## Abstract

Mining activities in arid regions of China have led to severe environmental degradation, including soil erosion, vegetation loss, and contamination of soil and water resources. These impacts are particularly pronounced in abandoned mining areas, where the cessation of mining operations has left vast landscapes unrehabilitated. In response, the Chinese government has implemented a series of legal and regulatory frameworks, such as the “Mine Environmental Protection and Restoration Program”, aimed at promoting ecological restoration in these areas. However, the unique environmental conditions of arid regions, including water scarcity, extreme temperatures, and poor soil quality, present significant challenges to restoration efforts. This review provides a comprehensive analysis of the ecological restoration of abandoned mining areas in China’s arid regions, focusing on the legal framework, restoration techniques, and evaluation systems. The restoration methods, their initiation timelines, monitoring systems, and the cost–benefit aspects of various strategies are critically reviewed alongside case studies from regions such as the Mu Us Desert and Qaidam Basin. Key strategies like phytoremediation, soil rehabilitation, and water resource management are assessed for their effectiveness, while challenges in enforcement, socioeconomic integration, and community engagement are discussed. This review concludes that while significant progress has been made, further improvements in restoration practices and evaluation systems are essential for long-term sustainability. Integration of socioeconomic indicators, community involvement, and advanced monitoring technologies are necessary for successful outcomes.

## Simple Summary

Abandoned mining areas in the arid regions of China face severe environmental challenges, including soil erosion, vegetation loss, and water contamination, which hinder ecological restoration efforts. This review explores strategies for optimizing vegetation restoration in these regions by analyzing legal frameworks, restoration techniques, and evaluation systems. Key methods such as phytoremediation, soil rehabilitation, and water management are assessed for their effectiveness. We highlight the importance of socioeconomic integration, community engagement, and advanced monitoring systems in achieving sustainable restoration outcomes. This summary aims to provide a concise and accessible overview of the study for a broader audience, emphasizing the critical need for improved restoration practices in these degraded landscapes.

## 1. Introduction

Mining has historically been a cornerstone of economic development in China, especially in resource-rich regions like Inner Mongolia, Xinjiang, and Gansu, where coal, metal ores, and other minerals have fueled rapid industrial growth [1,2]. However, the environmental costs have been profound, particularly in arid regions where ecosystems are fragile and sensitive to disruption due to harsh climatic conditions and limited water availability. Land degradation, loss of biodiversity, and contamination of soil and water resources are particularly pronounced in these regions, exacerbating issues like desertification and pollution [3,4].

In abandoned mining areas, operations often leave vast tracts of land without rehabilitation, posing long-term ecological risks. Without proper closure plans, the landscape remains degraded, and natural recovery processes are insufficient to restore ecological balance [5]. As a result, these areas are characterized by soil erosion, vegetation loss, and hydrological breakdown. Abandoned mines have far-reaching impacts, not only on the environment but also on local communities that rely on natural resources for their livelihoods [6].

To address these challenges, the Chinese government has implemented a range of policies, including the “Mine Environmental Protection and Restoration Program” (MEPRP), which mandates that mining companies develop restoration plans as part of their operating licenses [6]. These policies aim to promote ecological restoration, but arid regions’ unique environmental conditions make restoration difficult. Water scarcity, extreme temperatures, and poor soil quality hinder ecosystem recovery, requiring tailored restoration strategies that integrate ecological, hydrological, and engineering perspectives [7].

This review seeks to provide a comprehensive overview of the current state of ecological restoration in abandoned mining areas in China’s arid regions. It will examine the legal and regulatory frameworks governing these efforts. It will evaluate restoration techniques and methodologies and assess the effectiveness of existing evaluation systems. Additionally, the review will highlight successful case studies from regions like the Mu Us Desert and Qaidam Basin. This will offer practical insights into the restoration strategies implemented. This extended review will also address the timeline of restoration activities. It will also address the types of monitoring employed and provide a cost–benefit analysis of the approaches used, along with recommendations for future improvements.

## 2. Overview of the Abandoned Mining Area in Arid Region of China

### 2.1. Geographic and Climatic Characteristics

**Figure 1:**
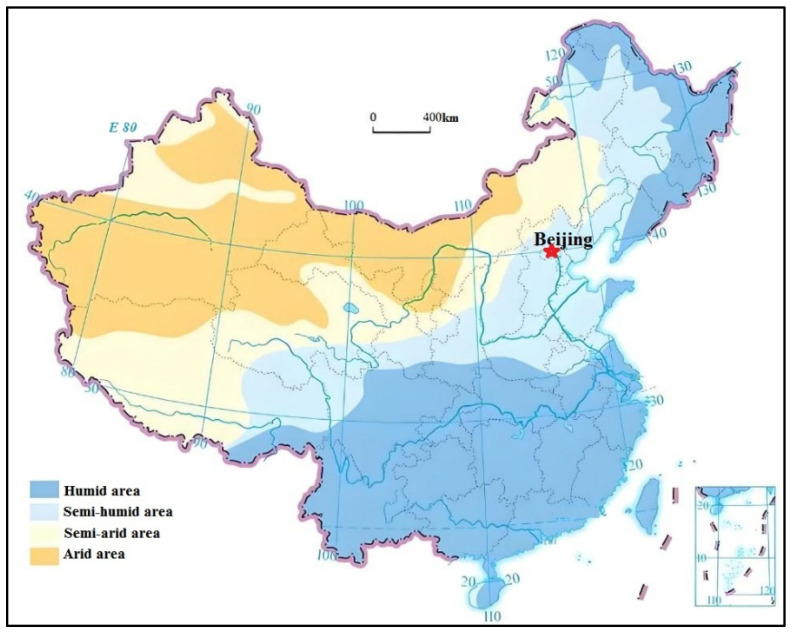
Distribution of arid and humid region of China (driving number of map: GS (2019) 1823).

The arid regions of China, covering provinces such as Xinjiang, Gansu, Inner Mongolia, and parts of Qinghai, are among the most ecologically sensitive areas in the country (Figure 1). These regions are characterized by harsh climatic conditions, including low annual precipitation (often less than 200 mm), high evaporation rates, and extreme temperature fluctuations between day and night [8]. Due to the limited rainfall and extreme climatic variability, vegetation in these areas is sparse and often highly specialized, adapted to withstand both drought and poor soil conditions. The soils in these regions, largely composed of sand and silt, are particularly prone to desertification and erosion when disturbed by human activities like mining [9].

Mining exacerbates the fragility of these ecosystems. The extraction of minerals—particularly coal, copper, and iron ore leads to the removal of the vegetative cover, exposing the soil to wind and water erosion. Moreover, mining tailings, which are often left uncovered, become sources of dust pollution. These tailings can also leach heavy metals into surrounding soils and water bodies, further aggravating environmental degradation [10]. The combination of climatic stressors and mining-related disturbances creates a unique set of ecological challenges in arid regions.

### 2.2. Extent of Abandoned Mining Areas

Mining in these arid regions has a long history, often stretching back several decades. These operations have primarily focused on extracting valuable minerals such as coal, copper, and gold, which are abundant but have been exploited with little regard for environmental consequences. Over the years, as mineral resources have been depleted, many of these mining sites have been abandoned without adequate closure plans, leaving behind a legacy of environmental degradation [11,12]. The current status of these abandoned mining areas is a testament to the intensive exploitation and inadequate post-mining land management practices. Many of these sites are plagued with open pits, unstable tailings, and large amounts of untreated mining waste [13]. The absence of vegetation and the presence of highly compacted soils have led to severe soil erosion, particularly during the rare but intense rainfall events typical of arid climates. This not only hinders natural vegetation re-establishment but also poses significant risks to nearby human settlements due to dust storms and flash flooding [14].

Abandoned mining areas in China’s arid regions represent a significant proportion of the total degraded land in the country. According to data from the Ministry of Natural Resources of China, over 13,000 hectares of land in arid regions have been impacted by mining activities, with approximately 70% of these areas abandoned due to the exhaustion of mineral resources or the closure of mining operations for economic or regulatory reasons [15]. These abandoned areas often lack viable natural vegetation and exhibit severe soil compaction and pollution from heavy metals, making natural recovery without human intervention virtually impossible [16].

The arid mining regions exhibit a range of abandoned landscapes, including open-pit mines, underground mines, and tailings ponds. Open-pit mining, which is the predominant method used in the extraction of coal and metallic ores in these regions, has left vast scars on the landscape, characterized by steep slopes, deep pits, and exposed soils [17]. Tailings ponds, which store the waste products of mineral processing, are sources of windborne dust, which spreads pollutants over large distances, further degrading the surrounding environment [18]. These sites, if left untreated, pose long-term risks to both local ecosystems and human health.

### 2.3. Environmental Impact of Mining Abandonment

**Figure 2:**
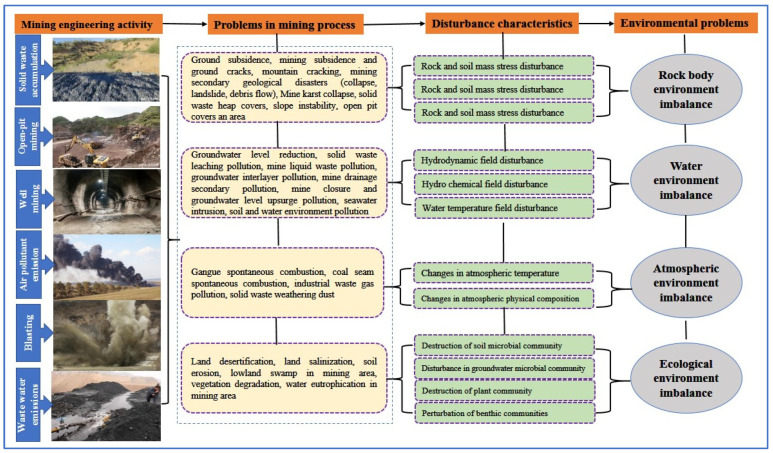
Environmental impact of mining activities.

The abandonment of mining operations in these arid regions has led to multiple intertwined environmental problems [19]. Key issues include soil degradation, loss of biodiversity, and hydrological disruptions (Figure 2).

#### 2.3.1. Soil Degradation

Mining activities strip away the topsoil, which contains the most fertile and biologically active layers. Once abandoned, the land suffers from significant soil erosion, worsened by the lack of vegetation cover and the harsh climatic conditions typical of arid zones [20]. Wind erosion is particularly rampant, with soil particles being transported over vast distances, often contributing to the problem of desertification, which is prevalent in arid regions. Studies have shown that the soil organic matter content in these areas is often reduced by more than 50%, severely compromising the land’s ability to support plant life [21].

#### 2.3.2. Vegetation Loss

The removal of native vegetation during mining operations creates barren landscapes that are difficult to rehabilitate. Natural revegetation processes are hindered by the arid climate, poor soil conditions, and the presence of heavy metals, which inhibit seed germination and plant growth [21]. Research indicates that less than 10% of abandoned mining areas in China’s arid regions have seen significant natural vegetation recovery over a 10-year period without active human intervention. This lack of vegetation further exacerbates soil erosion and reduces habitat availability for local wildlife, contributing to a decline in biodiversity [22].

#### 2.3.3. Hydrological Disruptions

Mining activities also disrupt the hydrological balance in these regions, primarily by altering the natural flow of surface water and groundwater [22]. The excavation of large pits and the removal of large quantities of earth can lead to changes in the water table, while the creation of tailings ponds and waste dumps can result in the contamination of nearby water sources with pollutants such as arsenic, mercury, and cadmium [23]. In arid regions, where water resources are already scarce, the contamination or depletion of water resources due to mining activities can have severe consequences for both human populations and local ecosystems [23].

### 2.4. Socioeconomic Impacts and Community Integration

Incorporating restoration projects into the community’s socioeconomic framework is essential, especially in economically disadvantaged regions where mining operations historically provided jobs [24]. Mine closure leads to unemployment and exacerbates poverty, necessitating community involvement in restoration activities. Communities play a key role in ensuring restoration projects’ long-term sustainability. Indicators such as employment generation, improvements in local agricultural productivity, and water access are crucial to measuring the success of these projects [25]. However, challenges remain in securing local communities’ participation, which may lack the technical expertise and resources to take part in these activities. Community participation has been improved through training programs in sustainable land management and financial incentives for restoration efforts. Nonetheless, additional work is needed to ensure more widespread and sustained community involvement [26].

### 2.5. Impacts on Local Communities and Biodiversity

Abandoned mining areas in arid regions degrade the physical environment but also affect regional communities and biodiversity. Mining operations closure typically leads to socioeconomic challenges, particularly in rural areas where local economies depend on the mining industry [27]. Loss of employment, decline in agricultural productivity due to soil degradation, and contamination of water resources are common issues that exacerbate poverty in these regions. Furthermore, environmental degradation associated with abandoned mines, such as air and water pollution, poses serious health risks to local populations, including respiratory diseases and exposure to toxic heavy metals [28].

Ecological impacts are equally severe, with native habitat destruction leading to biodiversity decline. The removal of vegetation and topsoil during mining operations results in habitat fragmentation and loss, disrupting local ecosystems and leading to native species decline or extirpation [29]. The lack of natural recovery processes in arid regions, where harsh climatic conditions impede vegetation re-establishment, further exacerbates biodiversity loss. Species adapted to arid environments, such as xerophytic plants and desert fauna, are particularly vulnerable to habitat destruction. Their populations have shown limited resilience in the face of such disturbances [30].

Restoration of degraded areas is essential not only for ecological recovery but also for supporting human livelihoods. It involves returning ecosystems to their original state or function, with a focus on conserving biodiversity and enhancing ecosystem services. However, restoration is not the only strategy for addressing land degradation [25]. Other approaches, such as reforestation, which involves planting trees to restore vegetation cover, and reclamation, which focuses on making degraded land productive or usable, are also critical alternatives [31]. These strategies are often context-specific and can complement restoration efforts depending on the type and severity of degradation. For example, reforestation contributes to carbon sequestration and habitat creation, while reclamation is particularly effective in industrial or mining landscapes [32].

Efforts to restore and manage degraded landscapes whether through restoration, reforestation, or reclamation—have demonstrated significant benefits, such as improved air and water quality, enhanced soil fertility, and local microclimate stabilization [32]. However, integrating local communities into these projects remains a critical challenge. To ensure both environmental and social benefits, socioeconomic indicators such as employment opportunities, agricultural productivity, and community health must be incorporated into the planning and implementation of these strategies [30].

## 3. Regulation and Achievements of Ecological Restoration of Damaged Mining Areas

### 3.1. Legal and Regulatory Framework

The regulatory landscape governing ecological restoration in China has evolved significantly over the past few decades, reflecting an increasing recognition of the importance of sustainable environmental management [33]. Key policies include the “Reclamation Act”, which mandates the restoration of mining sites to their original state or better. This act requires mining companies to submit reclamation plans for approval before the commencement of mining activities and to set aside funds for ecological restoration [34]. Additionally, the “Environmental Protection Law and the Law on the Prevention and Control of Desertification” provide a broad legal framework for the protection and rehabilitation of environments affected by mining. These laws emphasize the restoration of ecological balance, focusing on soil conservation, vegetation restoration, and water preservation [35].

In response to the environmental degradation caused by extensive mining operations, the Chinese government has implemented a series of legal and regulatory measures aimed at mitigating the environmental impacts and promoting ecological restoration [36]. The core of China’s regulatory framework on mining rehabilitation includes the “Environmental Protection Law” (2014), “Mineral Resources Law” (1996), and the “Land Rehabilitation Regulations” (1989). These laws outline the responsibilities of mining enterprises in mitigating environmental impacts and restoring damaged landscapes during and after mining operations [37].

In 2007, the government introduced the “Mine Environmental Protection and Restoration Program” (MEPRP), which requires mining companies to submit and execute a comprehensive restoration plan as a condition for their mining license [38]. Under this regulation, companies must invest in restoring ecosystems, reducing pollution, and rehabilitating soil quality after their mining operations cease. This has been supported by the “National Plan for Land Greening (2016–2020)”, which emphasizes large-scale afforestation and the recovery of ecological services in regions affected by mining [39].

Furthermore, financial instruments have been established to ensure the availability of resources for long-term restoration. For instance, mining enterprises are required to allocate funds to an environmental restoration fund, which is used to rehabilitate mining sites once operations conclude [40]. However, despite the comprehensive framework, enforcement remains a significant challenge, particularly in remote areas, where local governments often lack the resources or technical capabilities to implement and monitor restoration efforts effectively [41].

### 3.2. Ecological Restoration Techniques

**Figure 3:**
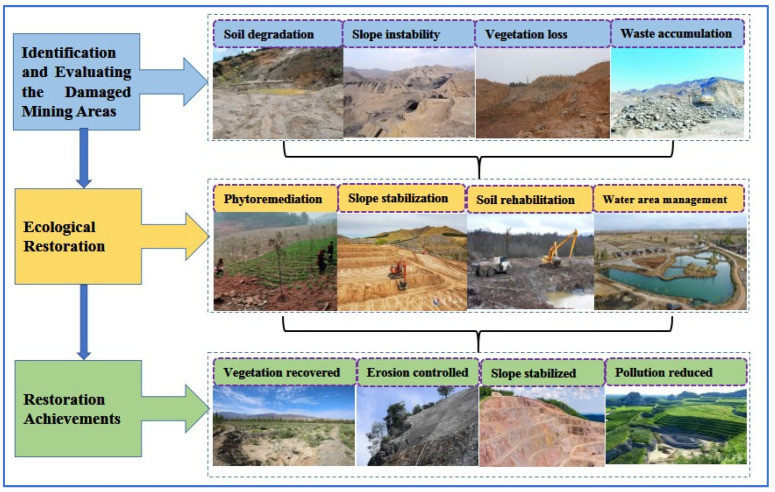
Workflow of ecological restoration in damaged mining areas.

China has adopted a variety of ecological restoration techniques to rehabilitate damaged mining areas [42]. These approaches are tailored to the specific characteristics of the region, including the type of mining activity, local soil conditions, and climate (Figure 3).

#### 3.2.1. Phytoremediation

Phytoremediation is one of the most widely used techniques for restoring vegetation in mining areas. This approach involves the planting of hyperaccumulator plants that can absorb heavy metals from contaminated soils, thereby reducing the levels of toxins in the ecosystem [43]. In China, species such as “*Artemisia capillaris*”, “*Phragmites australis*”, and “*Sedum plumbizincicola*” have been widely used for this purpose. Phytoremediation has shown promise in restoring the soil quality of mining areas by improving organic matter content and increasing biodiversity [44].

#### 3.2.2. Soil Rehabilitation

Soil degradation is one of the most critical challenges in the rehabilitation of abandoned mining areas. To address this, various soil amendments are applied to improve soil structure, fertility, and microbial activity [45]. These amendments include the addition of organic matter, such as compost or biochar, and the use of chemical agents to stabilize heavy metals. The application of biochar, for instance, has been shown to increase soil carbon content and reduce the bioavailability of toxic heavy metals, thereby enhancing the conditions for plant growth [46].

#### 3.2.3. Engineering and Structural Approaches

In areas where the topography has been severely altered by mining activities, engineering techniques are employed to stabilize slopes, prevent erosion, and create more suitable conditions for vegetation growth [47]. For example, terracing and the construction of retaining walls are commonly used to prevent landslides and minimize soil erosion on steep slopes. Additionally, tailings ponds, which pose a significant risk of windborne dust pollution, are often capped with a layer of impermeable material to prevent the spread of pollutants [48].

#### 3.2.4. Water Resource Management

In regions where mining activities have disrupted the local hydrology, water resource management techniques are employed to restore the natural flow of surface water and mitigate groundwater contamination [49]. These methods include the construction of drainage systems, wetlands, and water retention ponds to control runoff and filter contaminants. Wetlands, in particular, have proven effective in reducing heavy metal concentrations in water bodies near abandoned mining sites [50].

### 3.3. Achievements in Ecological Restoration

In recent years, significant progress has been made in the ecological restoration of abandoned mining areas in China’s arid regions. These achievements span various dimensions, including vegetation recovery, soil stabilization, pollution reduction, and water management [51]. The combined efforts of governmental policies, scientific research, and on-ground restoration practices have led to measurable improvements in the ecological health of previously degraded areas [52].

#### 3.3.1. Vegetation Recovery

One of the most prominent successes in restoration has been the re-establishment of vegetation in previously barren landscapes [53]. Large-scale afforestation and revegetation efforts have been implemented, utilizing drought-resistant and native plant species, particularly in regions like Inner Mongolia and Shanxi. Species such as *Caragana korshinskii* and *Hedysarum scoparium* have been employed due to their high tolerance to arid conditions and ability to stabilize soils [54]. According to reports from the Ministry of Natural Resources, over 10,000 hectares of degraded land have been successfully restored, with vegetation coverage improving significantly. Remote sensing data, such as Normalized Difference Vegetation Index (NDVI) measurements, show that restored areas have experienced increased biomass and biodiversity, indicating a successful re-establishment of plant communities [55].

#### 3.3.2. Soil Stabilization and Erosion Control

Soil degradation and erosion are critical challenges in abandoned mining areas, particularly in arid regions where soil fertility is already low. Achievements in this area include the application of soil amendments such as biochar, compost, and chemical fertilizers. These amendments have improved soil structure, increased organic matter, and enhanced microbial activity [56]. In addition to biological amendments, engineering solutions such as terracing and check dam construction have significantly reduced erosion rates. In some restored areas, soil erosion rates have been reduced by 40% compared to pre-restoration levels [57]. Soil stabilization has been particularly critical in areas with steep slopes and high wind erosion risk, such as the Mu Us Desert. In addition, soil organic matter content has increased, creating more favorable conditions for plant growth. In some areas, the introduction of leguminous plants, which fix atmospheric nitrogen, has further enhanced soil fertility [58].

#### 3.3.3. Pollution Reduction

Heavy metal contamination in soils and water bodies is one of the most severe environmental consequences of mining activities. Phytoremediation, the use of hyperaccumulator plants to remove heavy metals from soils, has been a key technique in reducing pollutant concentrations in restored areas [59]. Species such as *Sedum plumbizincicola* and *Phragmites australis* have been shown to significantly decrease concentrations of lead, cadmium, and arsenic in contaminated soils over 5–10 years [60]. Studies indicate that in some regions, soil heavy metal concentrations have decreased by more than 50%, contributing to ecosystem rehabilitation and reducing health risks to local communities [61,62].

### 3.4. Challenges and Areas for Improvement

Despite these successes, the journey toward effective ecological restoration has not been without challenges. Issues such as inadequate funding, limited technical knowledge, and lack of long-term monitoring and maintenance have occasionally hampered progress [63]. However, each challenge has provided valuable lessons that have refined restoration strategies and techniques. For instance, the importance of involving local communities in the restoration process has been recognized as crucial for ensuring the sustainability of restoration efforts [64].

While significant progress has been made in restoring damaged mining areas in China, several challenges remain [65]. First, the enforcement of restoration regulations is inconsistent, particularly in remote areas, where local authorities often lack the resources or expertise to monitor compliance. Additionally, the restoration of severely degraded soils, particularly those contaminated with heavy metals, remains a time-consuming and costly process [66].

Moreover, the ecological recovery of mining areas in arid regions, where water resources are limited, presents unique challenges. In these areas, the success of vegetation recovery is often hampered by insufficient rainfall and poor soil quality. Innovative techniques, such as the use of drought-resistant plant species and soil moisture retention strategies, need to be further developed and implemented [67].

Looking forward, China is focusing on integrating more innovative and science-based approaches into its restoration practices, such as the use of remote sensing technology for monitoring restored areas and the application of advanced biotechnology in vegetation restoration. Moreover, there is a growing emphasis on creating more inclusive policies that not only address environmental concerns but also promote economic development, thus aligning ecological goals with broader national development objectives.

### 3.5. Cost–Benefit Analysis and Timelines of Restoration Methods

Each restoration approach in China’s arid mining regions carries its own set of costs and benefits, which should be considered when planning and implementing rehabilitation projects [68]. Restoration methods such as phytoremediation and soil rehabilitation have varying timelines, with phytoremediation typically taking 5–10 years for significant pollutant reduction. In contrast, engineering approaches like terracing or tailings pond stabilization can provide more immediate but costly results [69].

#### 3.5.1. Timelines

Restoration processes, such as phytoremediation, typically begin during the early stages of rehabilitation, often starting within a year of site abandonment. However, phytoremediation is slow; significant improvement in soil contamination may take 5 to 15 years, depending on the severity of pollution and plant species selected [70]. In contrast, engineering approaches (e.g., terracing, slope stabilization, and tailings pond capping) often deliver immediate improvements but at a much higher upfront cost. These interventions may take only 1–2 years to implement but require continuous maintenance to remain effective [71]. For example, projects initiated in the Mu Us Desert began over 20 years ago, and monitoring has revealed both successful outcomes and areas needing additional intervention [72].

#### 3.5.2. Monitoring Systems

Monitoring has been inconsistently applied across restoration sites, with varying evaluation and monitoring durations. In well-documented cases, remote sensing tools, such as satellite imagery and UAVs, have been integrated with on-the-ground soil and vegetation sampling to track recovery progress [73]. However, many projects lack standardized long-term monitoring protocols, which are essential for evaluating restoration success. In several instances, monitoring systems were established for only short periods, typically ranging from 2 to 5 years, and the absence of extended monitoring data has hindered efforts to assess the full impact of restoration interventions [74].

To ensure the effectiveness of restoration projects, consistent monitoring over periods of at least 10 to 15 years is recommended. This allows for the collection of sufficient data to evaluate long-term ecological recovery and sustainability. Adaptive management should be implemented more extensively, enabling restoration methods to be refined and adjusted based on feedback from continuous, long-term monitoring [75].

#### 3.5.3. Cost–Benefit Considerations

Restoration strategies must carefully balance financial costs with ecological and socioeconomic benefits [76]. Different approaches vary widely in terms of implementation expenses, speed of outcomes, and long-term effectiveness. For instance, phytoremediation, while initially cheaper, requires ongoing management and monitoring to ensure effective pollutant extraction and plant growth. In contrast, soil amendments such as biochar entail higher upfront costs but offer faster improvements in soil fertility and structural stability, making them a preferred choice for heavily degraded soils [77].

Engineering solutions, such as retaining walls or gabions, provide immediate erosion control but demand significant capital investment and may not always align with ecological objectives. Similarly, artificial wetlands deliver substantial benefits, including water treatment and habitat restoration, but are only viable in regions with adequate water availability to sustain such systems [78]. Conversely, in arid regions like the Qaidam Basin, a combination of soil amendments and water-saving irrigation techniques has proven more cost-effective, successfully supporting plant re-establishment under challenging conditions [79].

To facilitate a clearer understanding of the trade-offs involved in restoration efforts, Table 1 presents a comparative cost–benefit analysis of common restoration strategies. This evaluation underscores the importance of tailoring restoration methods to local environmental and socioeconomic contexts to maximize both efficiency and impact.

### 3.6. Critical Comparison of Restoration Processes and Region-Specific Approaches

The choice of restoration method is heavily influenced by local environmental conditions and resources [80]. For example, in the Mu Us Desert, afforestation efforts focused on *Caragana korshinskii* and *Hedysarum scoparium*, species adapted to low rainfall and poor soils [81], while in the Qaidam Basin, drip irrigation systems were favored due to extreme water scarcity [82]. Artificial wetlands have been deployed to manage water pollution in areas like Shanxi, but these would be inappropriate in drier regions without sufficient water supply [83].

Each approach was selected based on relevance to the site’s conditions. For example, the slow natural recovery rate in the Qaidam Basin led to water-efficient techniques [84]. In contrast, afforestation in the Mu Us Desert was prioritized to combat soil erosion and stabilize sand dunes, addressing the primary ecological threat in that region [85].

### 3.7. Financial and Material Considerations in Rehabilitation

Rehabilitation of degraded territories, particularly in arid regions impacted by mining, requires substantial financial investments. The costs can vary significantly depending on the extent of the damage, the chosen restoration strategy, and the local environmental conditions [36]. On average, rehabilitation projects can cost between USD 500 and USD 5000 per hectare, with the variation largely depending on the complexity of the restoration efforts. The primary costs associated with these projects typically involve site preparation, material procurement, labor, and long-term monitoring [61].

Site preparation is often one of the most expensive phases, especially if the land has been severely degraded. It includes land clearing, soil conditioning, and infrastructure development, which can be particularly costly for regions with significant degradation [73]. The materials used in rehabilitation projects vary depending on the specific restoration method employed. Common materials include soil amendments such as biochar or compost, seeds for vegetation restoration, and, in some cases, engineered solutions like geotextiles or erosion control products. For example, the application of biochar may cost between USD 300 and USD 1000 per hectare, while the cost of seeding can range from USD 50 to USD 500 per hectare. The choice of material depends on the degradation level of the soil and the desired restoration outcome [80,83].

Labor costs are another significant component of rehabilitation projects. A considerable amount of human resources is required for planting, monitoring, and maintaining the restored area. Labor costs generally account for 30–40% of the total budget, and these costs can vary depending on the region [86]. In addition to labor, long-term monitoring and maintenance are crucial for the success of rehabilitation efforts. This includes regular soil testing, vegetation health assessments, and maintenance activities such as irrigation. These ongoing costs can amount to 10–20% of the initial investment annually [87].

The volume of materials used in rehabilitation depends on the scale of the project and the specific restoration techniques employed. For example, the application of soil amendments, such as biochar or compost, typically ranges from 50 to 200 tons per hectare, depending on the soil’s degradation level [88]. When restoring vegetation, 20–50 kg of native seeds per hectare are commonly used, although this can vary based on plant species and the chosen seeding method. In arid regions, water and irrigation systems represent a substantial portion of the total rehabilitation costs, often making up 20–30% of the overall budget. For large-scale projects, the cost of irrigation systems can range from USD 500 to USD 2000 per hectare, depending on the irrigation method selected (e.g., drip irrigation or sprinklers) [89,90,91,92].

## 4. Evaluation System of Damaged Mining Areas in China

### 4.1. Soil Degradation Evaluation

#### 4.1.1. Soil Physical Properties

Soil degradation is one of the primary indicators used to assess the environmental impact of mining activities. The evaluation of soil degradation in damaged mining areas primarily involves analyzing changes in soil physical properties such as soil structure, compaction, porosity, and erosion rates [86]. Mining operations often lead to the removal of the topsoil layer, which contains the majority of soil organic matter and microorganisms that are vital for maintaining soil fertility and structure. Consequently, the remaining soil becomes compacted and less porous, leading to reduced infiltration rates and increased susceptibility to erosion [88].

Soil compaction is measured by soil bulk density and penetration resistance. These parameters reflect the extent to which soil has been compacted by heavy machinery used during mining activities [89]. A bulk density of over 1.6 g/cm^3^ in these regions typically indicates poor soil structure and a reduced capacity for water retention and root penetration. In some severely impacted areas, bulk density values can exceed 2.0 g/cm^3^, rendering the soil almost impermeable and inhibiting vegetation recovery [90].

In addition, soil erosion rates are commonly measured using erosion pins and surface runoff measurements. The steep slopes and exposed soil surfaces in mining areas lead to higher erosion rates, particularly in arid regions where wind erosion is a major concern [91]. Studies have shown that soil erosion rates in some abandoned mining sites can exceed 100 t/ha/year, which is significantly higher than the natural erosion rates in undisturbed ecosystems [92].

#### 4.1.2. Soil Chemical Properties

Another critical aspect of soil degradation is the alteration of soil chemical properties, particularly in terms of soil pH, nutrient content, and the presence of toxic elements such as heavy metals [93]. Mining activities, especially those involving metal ores, often lead to the contamination of soils with hazardous substances such as lead (Pb), cadmium (Cd), and arsenic (As). These elements not only degrade soil quality but also pose long-term risks to human health and local ecosystems [94].

The evaluation of soil contamination involves measuring the concentrations of heavy metals in the soil and comparing them to national environmental standards. In China, the “Soil Environmental Quality Standard” (GB 15618-2018) sets the permissible levels for various heavy metals [95]. For example, the standard allows for a maximum concentration of 80 mg/kg for lead in agricultural soils, whereas concentrations in abandoned mining areas often exceed 300 mg/kg, indicating severe contamination [96].

In addition to heavy metals, soil nutrient content is evaluated by measuring organic matter, nitrogen, phosphorus, and potassium levels. Mining activities reduce soil organic matter content, leading to a loss of soil fertility. Studies have shown that in severely degraded mining areas, soil organic matter content can be reduced by more than 60%, further hampering vegetation recovery efforts [97].

### 4.2. Vegetation Damage Evaluation

#### 4.2.1. Vegetation Coverage

Vegetation coverage is a key indicator of ecosystem health and recovery in damaged mining areas. The loss of vegetation during mining operations significantly alters local ecosystems, and the re-establishment of vegetation is one of the primary goals of ecological restoration. Evaluating vegetation damage involves analyzing satellite imagery, drone surveys, and field measurements to assess changes in vegetation cover over time [98].

In China, vegetation damage is commonly assessed using the Normalized Difference Vegetation Index (NDVI), which measures the density of green vegetation on the land surface. NDVI values range from −1 to +1, with higher values indicating healthier vegetation. In abandoned mining areas, NDVI values are often below 0.2, indicating sparse or non-existent vegetation [99]. By contrast, restored areas where vegetation has successfully recovered typically exhibit NDVI values above 0.5, demonstrating significant ecological recovery.

#### 4.2.2. Species Diversity and Composition

Beyond simple coverage, evaluating the species diversity and composition of vegetation is crucial for understanding the ecological impact of mining activities. Mining can lead to the displacement of native plant species and the proliferation of invasive species, which are often less effective at stabilizing soil and supporting biodiversity [100]. Field surveys are conducted to measure species richness (the number of different plant species) and species evenness (the distribution of individuals among species) in both degraded and restored areas [101].

Studies in China’s mining regions have shown that species richness in abandoned mining sites is typically 30–50% lower than in undisturbed areas, and native species are often outcompeted by invasive plants such as “*Atriplex canescens*” and “*Salsola tragus*”, which are more tolerant of poor soil conditions [102]. Restoring native species diversity is a key goal in ecological restoration, as higher diversity increases ecosystem resilience and supports a wider range of fauna.

### 4.3. Environmental Pollution Evaluation

#### 4.3.1. Air Pollution

Air pollution, particularly dust pollution from tailings and unrehabilitated mining areas, is a significant environmental concern. Dust generated by wind erosion or mining activities can contain harmful particles, including heavy metals that pose serious health risks to local populations and contribute to regional air quality degradation [103]. Airborne particles are commonly assessed using particulate matter (PM) measurements, with PM10 and PM2.5 being the most relevant sizes for human health. Studies have found that PM10 concentrations in some mining areas can exceed 150 µg/m^3^ [104,105], which is far above the World Health Organization’s recommended safe levels (WHO, 2019).

Moreover, the presence of heavy metals in dust particles significantly increases the toxicity of airborne pollutants. The concentrations of metals such as lead and cadmium in mining dust often exceed safe levels, contributing to a higher incidence of respiratory diseases in nearby communities [45].

#### 4.3.2. Water Pollution

Water pollution is another critical concern in the evaluation of damaged mining areas, particularly in terms of the contamination of surface water and groundwater with toxic substances. Mining activities often lead to the leaching of heavy metals and other pollutants into nearby water bodies, which can have severe ecological and health impacts [106].

The evaluation of water pollution in mining areas involves the analysis of water samples for contaminants such as heavy metals, sulfates, and acidity. In China, the “Environmental Quality Standards for Surface Water” (GB 3838-2002) outlines permissible levels for various pollutants [107]. For instance, the maximum allowable concentration of arsenic in drinking water is set at 0.01 mg/L, while levels in mining-affected areas can exceed 0.05 mg/L, posing significant risks to human health and aquatic ecosystems [108].

In addition to chemical contamination, the physical characteristics of water, such as turbidity and sediment load, are also important indicators of mining-induced water pollution. Elevated sediment loads can smother aquatic habitats and disrupt the natural flow of rivers, further degrading the ecosystem [109].

### 4.4. Method for Evaluation

#### 4.4.1. Commonly Used Evaluation Methods

To systematically assess the criteria mentioned above, a variety of methods are employed, including the following:

Remote Sensing and GIS Technologies: These tools are instrumental in tracking changes over large areas and over time, providing valuable data on land cover changes, vegetation health, and erosion.

Ecological Indices: Various ecological indices, such as the Soil Quality Index (SQI) and the Vegetation Condition Index (VCI), are used to quantify the extent of ecological recovery or ongoing degradation.

Community-Based Assessments: Involving local communities in the evaluation process helps gather unique insights into the socioeconomic impacts of mining and the effectiveness of restoration efforts.

#### 4.4.2. Limitations of Current Evaluation Systems

While the existing evaluation systems are robust, they do face limitations. These can include the high cost and technical complexity of some assessment methods, potential biases in remote sensing data, and the difficulty in obtaining timely and accurate data from remote or inaccessible areas. Additionally, there is often a lack of integration between different types of data, which can hinder comprehensive assessments.

To enhance the effectiveness of evaluation systems, several recommendations can be proposed:

Integration of Data Sources: Combining data from remote sensing, ground-based observations, and community feedback can provide a more holistic view of the environmental status and trends.

Development of Standardized Protocols: Establishing standardized protocols for data collection, analysis, and reporting can improve the consistency and comparability of environmental assessments across different regions.

Adoption of New Technologies: Emerging technologies, such as drones for aerial surveys and machine learning for data analysis, can significantly enhance the accuracy and efficiency of environmental evaluations.

### 4.5. Social Desertification Processes and Community Integration

#### 4.5.1. Social Desertification

Local communities decline due to environmental degradation and economic displacement, which has been a critical challenge in abandoned mining areas. As mining operations cease, jobs disappear, and poverty worsens, especially in marginalized areas [110]. The success of ecological restoration depends heavily on community integration and involvement in the restoration process. However, there have been notable failures in engaging local populations, particularly in areas where restoration plans were developed, without considering the socioeconomic realities of the region [111].

#### 4.5.2. Indicators of Community Integration

Community integration indicators include job creation through reforestation projects, incentives for sustainable agriculture, and improvements in access to water resources [99]. For instance, in the Ningxia Hui Autonomous Region, local farmers were employed in tree planting efforts and received financial incentives to participate in soil conservation activities. While these initiatives have shown success in some regions, community engagement remains limited due to a lack of technical expertise and insufficient financial support [76].

#### 4.5.3. Failures and Challenges

In some cases, restoration projects have not accounted for local livelihood needs. For example, tree planting initiatives may improve vegetation cover but fail to provide economic benefits to local populations if the species planted are not useful for fodder or timber [85]. The challenge lies in selecting restoration methods that not only improve ecological outcomes but also alleviate poverty and create sustainable livelihoods for the affected populations [110].

While China has made significant strides in the ecological restoration of abandoned mining areas, much more attention is needed to developing region-specific approaches that take into account a variety of ecological conditions, monitoring timelines, cost–benefit trade-offs, and community integration [112]. Restoration efforts that ignore socioeconomic factors risk alienating local populations and reducing long-term sustainability. Monitoring systems must be strengthened, and adaptive management techniques should be employed more consistently. Additionally, lessons learned from failures in community integration must be used to inform future projects, ensuring that local populations benefit both environmentally and economically from restoration initiatives [113].

#### 4.5.4. Challenges in Monitoring Socioeconomic Impacts

While many restoration efforts focus on environmental recovery, monitoring socioeconomic impacts has been less systematic. Indicators such as increased agricultural productivity and improved local employment rates should be incorporated into project evaluations to ensure that ecological restoration efforts contribute to the socioeconomic resilience of the region [114,115].

## 5. Recommendation for the Improvement of the Evaluation System and Practical Experience for Ecological Restoration of Damaged Mining Areas

### 5.1. Enhancing the Comprehensive Evaluation Criteria

#### 5.1.1. Integration of Ecological and Socioeconomic Indicators

**Figure 4:**
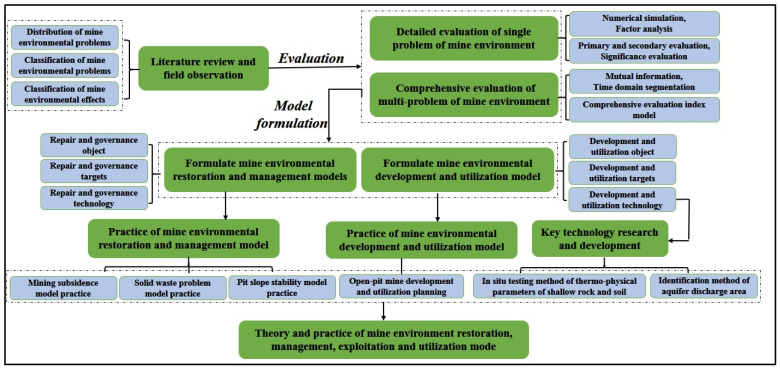
Application of restoration indexes and methods.

One of the key recommendations for improving the evaluation system of damaged mining areas is the integration of both ecological and socioeconomic indicators into a comprehensive framework. While the current evaluation systems primarily focus on environmental factors such as soil degradation, vegetation loss, and pollution levels, they often overlook the socioeconomic impacts of mining abandonment [116]. Mining areas are frequently located in economically disadvantaged regions, and the success of ecological restoration efforts should also be measured by their contribution to improving local livelihoods and community well-being (Figure 4).

To address this, future evaluation systems should include indicators such as job creation, improved agricultural productivity, and enhanced access to clean water and air. These socioeconomic factors are critical for the long-term sustainability of restoration projects and should be incorporated alongside traditional ecological metrics [117]. For example, the recovery of vegetation should not only be assessed based on coverage but also on its potential to provide economic benefits to local communities through sustainable agriculture or ecotourism [118].

#### 5.1.2. Development of a Region-Specific Evaluation Model

China’s arid regions exhibit unique environmental challenges, including low precipitation, high evapotranspiration rates, and severe desertification. As a result, a one-size-fits-all approach to the evaluation of ecological restoration is insufficient. The evaluation system should be tailored to the specific conditions of the arid regions, taking into account factors such as water scarcity, soil salinity, and the presence of drought-tolerant plant species [119].

The development of a region-specific evaluation model should involve the collaboration of ecologists, hydrologists, and soil scientists to create criteria that reflect the unique ecological dynamics of these areas. For instance, in regions with limited water resources, the success of vegetation recovery should be measured not only by overall coverage but also by the water-use efficiency of the restored ecosystems [120]. Additionally, soil restoration efforts should focus on improving soil moisture retention and combating salinization, which are critical factors in the success of restoration projects in arid climates.

### 5.2. Strengthening Monitoring and Enforcement Mechanisms

#### 5.2.1. Real-Time Monitoring Through Remote Sensing Technology

The current evaluation system for mining restoration in China relies heavily on periodic field surveys, which are often labor-intensive and resource-consuming. To improve the efficiency and accuracy of the evaluation process, it is recommended that real-time monitoring through remote sensing technologies be expanded [121]. Remote sensing tools, such as satellite imagery, unmanned aerial vehicles (UAVs), and ground-based sensors, can provide up-to-date data on vegetation coverage, soil conditions, and pollutant levels across large areas, reducing the need for frequent on-site inspections [122].

For instance, remote sensing can be used to monitor changes in vegetation cover through the use of indices like the Normalized Difference Vegetation Index (NDVI). These technologies allow for continuous tracking of restoration progress and can provide early warnings of potential failures in restoration efforts, such as the re-emergence of erosion or pollution [123]. Additionally, the integration of remote sensing data with Geographic Information Systems (GIS) can help local authorities to better plan and manage restoration activities by identifying areas that require urgent intervention [55].

#### 5.2.2. Enforcement of Environmental Regulations

While China has developed a robust legal framework for the restoration of damaged mining areas, enforcement remains a challenge, particularly in remote regions where local governments often lack the capacity or resources to ensure compliance. Strengthening the enforcement of environmental regulations is essential for the success of restoration projects [124].

One approach to improving enforcement is the establishment of third-party auditing mechanisms, where independent environmental agencies assess the compliance of mining companies with restoration requirements. These agencies can provide unbiased evaluations and ensure that companies fulfill their legal obligations to restore damaged sites [67]. Moreover, the creation of a transparent public reporting system, where the results of restoration audits are made publicly available, can increase accountability and incentivize companies to invest in more effective restoration practices.

### 5.3. Practical Experience in Ecological Restoration in Arid Regions

#### 5.3.1. Case Study: Vegetation Restoration in the Mu Us Desert

The Mu Us Desert, located in the northwestern part of China, represents one of the most successful examples of large-scale ecological restoration in arid mining regions. Over the past two decades, the Chinese government, in collaboration with local communities, has implemented extensive afforestation programs to restore vegetation in this highly degraded landscape [125]. The restoration efforts have primarily focused on planting native, drought-tolerant species, such as “*Caragana korshinskii*” and “*Hedysarum scoparium*”, which are well-adapted to the harsh climatic conditions of the region [126].

One of the key lessons from the Mu Us Desert restoration project is the importance of selecting plant species that are not only ecologically suitable but also economically valuable to local communities [127]. The introduction of leguminous shrubs, for example, has improved soil fertility by enhancing nitrogen fixation while also providing fodder for livestock, thus offering dual benefits for both ecological restoration and local livelihoods [128].

#### 5.3.2. Water Management in the Qaidam Basin

The Qaidam Basin in Qinghai Province is another area where practical experience in water management has significantly contributed to the success of ecological restoration projects. The region, characterized by its arid climate and saline soils, has implemented an innovative water-saving irrigation system to support vegetation recovery. This system, known as “drip irrigation”, delivers water directly to the roots of plants, minimizing evaporation losses and ensuring efficient use of scarce water resources [54,55].

Moreover, the Qaidam Basin project has integrated the use of artificial wetlands to treat wastewater from nearby mining operations. These constructed wetlands filter heavy metals and other pollutants from the water, reducing the environmental impact of mining activities and providing a sustainable water source for irrigation [129]. This approach has not only improved water quality but also supported the re-establishment of native plant species, which are essential for stabilizing the soil and reducing erosion.

### 5.4. Promoting Community Involvement in Restoration Projects

One of the most important factors in the long-term success of ecological restoration projects is the active involvement of local communities. In many cases, restoration efforts have failed because they did not take into account the needs and priorities of local populations, leading to a lack of support or even active resistance [56,57]. To address this issue, future restoration projects should prioritize community engagement from the planning stages through to implementation and monitoring.

For example, in the Ningxia Hui Autonomous Region, local farmers have been encouraged to participate in afforestation projects by providing financial incentives and training in sustainable land management practices [130]. By involving communities in restoration activities, not only is the success of the projects improved, but the socioeconomic benefits also help to alleviate poverty and promote sustainable development in these areas [131].

## 6. Conclusions and Future Recommendations

Ecological restoration of abandoned mining areas in arid regions of China has made substantial progress through targeted restoration techniques and comprehensive evaluation systems. This review highlights key achievements, including significant improvements in vegetation recovery, soil stabilization, and pollution reduction. Techniques such as phytoremediation, soil amendments, and innovative water resource management strategies have proven effective in addressing unique environmental challenges in arid regions. These regions are characterized by extreme temperatures, limited water availability, and poor soil quality. However, despite these advancements, significant challenges remain. The inconsistent enforcement of restoration policies, particularly in remote regions, coupled with limited technical expertise at the local level, continues to hinder the full success of these initiatives. Furthermore, heavy metal contamination in soils requires ongoing research to develop more effective and sustainable remediation technologies. Financial constraints and sustained investment also present persistent barriers to large-scale restoration success.

To enhance ecological restoration effectiveness in arid regions, future efforts should focus on several key areas. One of the most significant aspects of future restoration projects is the integration of socioeconomic factors into the planning and implementation stages. Engaging local communities in restoration activities is essential for long-term sustainability. Restoration projects should not only focus on environmental recovery but also consider job creation, improved agricultural productivity, and access to clean water and resources for local populations. Socioeconomic indicators should be integrated into evaluation frameworks to ensure that restoration benefits extend beyond ecological gains and improve individual livelihoods.

Advanced monitoring technologies, such as remote sensing, Geographic Information Systems (GIS), and unmanned aerial vehicles (UAVs), also need to be adopted more widely. These technologies can improve the accuracy and timeliness of restoration assessments by allowing for real-time monitoring of vegetation recovery, soil conditions, and pollutant levels across vast areas. Incorporating these tools into the restoration process will facilitate more adaptive management strategies, enabling timely interventions in response to environmental changes. Furthermore, strengthening ecological restoration regulations enforcement remains crucial. This can be achieved by developing third-party audit mechanisms and increasing transparency through public reporting of restoration efforts. Local governments also need financial and technical support to enforce regulations effectively, particularly in remote and economically disadvantaged regions.

Given the diverse environmental conditions of China’s arid regions, future research should focus on developing region-specific restoration models. A one-size-fits-all approach is inadequate, so restoration methods must be tailored to local climatic conditions, water availability, and soil characteristics. For example, selecting drought-resistant plant species or optimizing water-use efficiency will be key to restoration success. In addition, further research into advanced soil remediation technologies is necessary, particularly for addressing heavy metal contamination. The development of more efficient phytoremediation species, soil amendments, and bioremediation techniques should be prioritized. Combining these biological approaches with engineering solutions, such as soil stabilization and pollution containment structures, will provide a more comprehensive strategy for mitigating contamination.

Ecological restoration projects require ongoing maintenance and monitoring. Future research should explore cost-effective and sustainable approaches to maintaining restored ecosystems, especially in regions with harsh climates. Identifying low-maintenance plant species and sustainable agricultural practices that align with local environmental conditions will be crucial for lasting success. In conclusion, while significant strides have been made in the ecological restoration of abandoned mining areas in China’s arid regions, continuous efforts are needed to refine restoration techniques. These efforts are required to enhance regulatory enforcement and integrate community involvement and ecological interests. These efforts are essential not only for restoring ecological function but also for promoting regional development and improving local communities’ well-being in these fragile ecosystems. Achieving long-term success will depend on aligning restoration goals with socioeconomic needs and deploying innovative technologies to adapt to evolving environmental conditions.

## Figures and Tables

**Table 1:** Cost–benefit comparison of restoration strategies.

Restoration Strategy	Costs	Benefits
Ecological Restoration	High initial investment; ongoing monitoring costs; requires skilled labor.	Long-term recovery of ecosystem services (e.g., biodiversity, water quality); enhances climate resilience.
Reforestation	Moderate costs for seedlings, planting, and maintenance.	Carbon sequestration; habitat creation; reduced soil erosion; potential for timber production.
Reclamation	High costs for engineering interventions and soil amendments.	Immediate usability of degraded land for agriculture, industry, or recreation; reduces public health risks.

## Data Availability

Data may be provided upon reasonable request from the first author.
